# Role of Interaction Range and Buoyancy on the Adhesion of Vesicles

**DOI:** 10.1021/acs.langmuir.3c02715

**Published:** 2024-02-06

**Authors:** Lucia Wesenberg, Marcus Müller

**Affiliations:** Institute for Theoretical Physics, Georg-August University, Friedrich-Hund-Platz 1, 37077 Göttingen, Germany

## Abstract

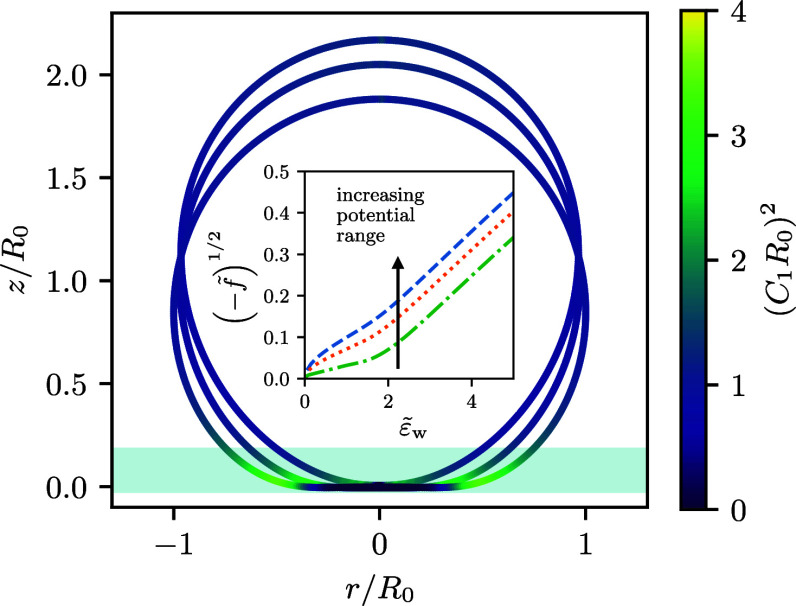

Vesicles on substrates play a fundamental role in many biological processes, ranging from neurotransmitter release at the synapse on small scales to the nutrient intake of trees by large vesicles. For these processes, the adsorption or desorption of vesicles to biological substrates is crucial. Consequently, it is important to understand the factors determining whether and for how long a vesicle adsorbs to a substrate and what shape it will adopt. Here, we systematically study the adsorption of a vesicle to planar substrates with short- and long-range interactions, with and without buoyancy. We assume an axially symmetric system throughout our simulations. Previous studies often considered a contact potential of zero range and neutral buoyancy. The interaction range alters the location and order of the adsorption transition and is particularly important for small vesicles, e.g., in the synapse. Whereas even small density differences between the inside and the outside of the vesicle give rise to strong buoyancy effects for large vesicles, e.g., giant unilamellar vesicles, as buoyancy effects scale with the fourth power of the vesicle size. We find that (i) an attractive membrane-substrate potential with nonzero spatial extension leads to a pinned state, where the vesicle benefits from the attractive membrane-substrate interaction without significant deformation. The adsorption transition is of first order and occurs when the substrate switches from repulsive to attractive. (ii) Buoyancy shifts the transversality condition, which relates the maximal curvature in the contact zone to the adhesion strength and bending rigidity, up/downward, depending on the direction of the buoyancy force. The magnitude of the shift is influenced by the range of the potential. For upward buoyancy, adsorbed vesicles are at most metastable. We determine the stability limit and the desorption mechanisms and compile the thermodynamic data into an adsorption diagram. Our findings reveal that buoyancy, as well as spatially extended interactions, are essential when quantitatively comparing experiments to theory.

## Introduction

The adsorption behavior of vesicles is critical in many biological processes, such as the transport of neurotransmitters between neurons or endo- and exocytosis in cells. In all of these processes, as well as in the drug release from a transport vesicle, the adsorption of vesicles to biological substrates and the concomitant dwell time, until leaving the substrate, is crucial.^1−3^ The main determinants of the adsorption behavior aside from the adsorption strength are bending energy and tension of the membrane. The tension of a membrane, however, is a nonintrinsic property and is determined by the configuration of the vesicle, stretching the membrane. It is thus of special interest to investigate when the shape of a vesicle is bending-energy or tension-dominated.

**Figure 1:**
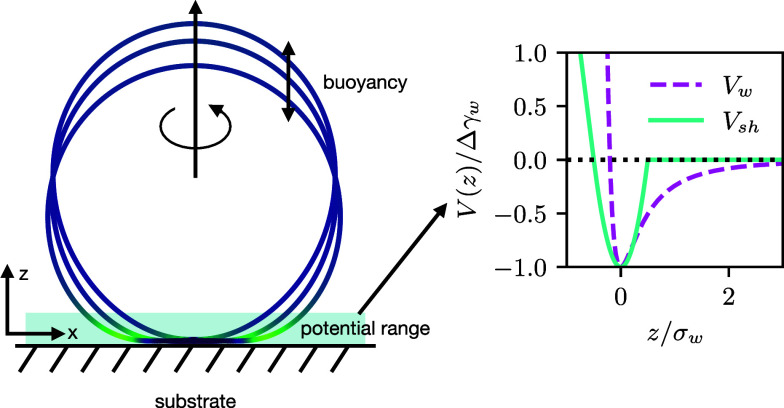
This study investigates axially symmetric vesicles in a spatially extended membrane-substrate potential, *V*_w_, with and without buoyancy.

Many studies have investigated the adsorption behavior of vesicles, both by theory and simulations, as well as experiments.^4−14^ However, the spatial extent of the interaction potential and buoyancy—two aspects, which are important for quantitative comparison to experimental data—have commonly been ignored. Previous analytical and numerical studies of static, adsorbed vesicles usually split the vesicle into two areas: (i) a planar adhesion zone, which does not bend and exclusively contributes to the energy via an attractive membrane-substrate interaction of zero range (contact interaction) and (ii) the remaining surface of the vesicle, where only the bending energy, proportional to the bending rigidity, κ, adds to the energy. The balance between adhesion and bending energies is then quantified by the dimensionless variable , with Δγ_w_ being the adhesion strength and *R*_0_ denoting the radius of the spherical, nonadsorbed vesicle, respectively. Here, we focus on two experimentally relevant extensions of this basic model—a nonzero spatial extent of the membrane-substrate interaction and buoyancy. The studied system is illustrated in Figure 1.

In all experiments, however, the range of the interaction between membrane and substrate has a nonzero spatial extent. Such a nonzero spatial extent of the membrane-substrate interaction gives rise to a “pinned” state where the vesicle may benefit from the attraction without deformation.^12,15^ This is especially relevant for small vesicles, such as, e.g., synaptic vesicles, where the range of the membrane attraction is not much smaller than the vesicle radius. Here, we demonstrate that a nonzero spatial extent of the membrane-substrate interaction will qualitatively alter the thermodynamics of adhesion, both the location of the transition and its dependence on vesicle size as well as its order.

Buoyancy is often used in experiments to initiate contact between vesicle and substrate. Already minuscule density differences between the interior of the vesicle and its surroundings give rise to sedimentation of the vesicle to the substrate. However, the effect of buoyancy on the vesicle shape has only rarely been considered.^7^ Previous work has shown that the balance between buoyancy and bending energy is characterized by the dimensionless characteristics  that scales with *R*_0_^4^. Thus, small density differences exert a significant impact on large vesicles. The process of vesicle sedimentation due to downward buoyancy has been investigated by experiment^16^ and in simulation.^17^ Prior work^7^ also considered the case of conserved enclosed volume, resulting in nonaxisymmetric vesicle shapes that may resemble that of red blood cells. These shapes lack rotational symmetry with respect to the substrate normal in their adsorbed state. In our work, we allow the vesicle volume to equilibrate and only study rotationally symmetric shapes.

We quantify how buoyancy as well as the spatial extent of the membrane-substrate interaction^18^ modify the transversality condition, ,^4−6^ that linearly relates adhesion strength and the square of the contact or maximal curvature, *C*_1max_. This relation is often employed in experiments to infer the adhesion strength.^18^ To describe the thermodynamics of vesicle adsorption, we borrow concepts from the wetting of liquids.^19−23^ In this analogy, we identify the center-of-mass position, *z*_cm_, of the vesicle with the thickness, *z*_int_, of the laterally uniform wetting layer. The dewetted state, where *z*_int_ < ∞ is microscopic, corresponds to a pinned or adhered vesicle, whereas the unbound vesicle is the analog of a macroscopically thick wetting film. We show that vesicle desorption is the analog of wetting with a repulsive short-range contribution to the interface potential and a long-range contribution that switches from attractive to repulsive at the first-order transition. Within this analogy, downward buoyancy, , corresponds to an undersaturated vapor and the vesicle is always in contact with the substrate. However, upward buoyancy, , is the analog of a supersaturated vapor, and the adhered vesicle is at best metastable. We summarize the thermodynamics in an adsorption diagram that describes the stability of different vesicle states as a function of dimensionless adhesion strength and buoyancy. This includes the limit of metastability (spinodal) of adsorbed vesicles for upward buoyancy that we extrapolate from the minimum energy path (MEP)^24−27^ of the vesicle-desorption process.

Our manuscript is arranged as follows: after introducing our model, we discuss the influence of the spatial extent of the membrane-substrate potential on the thermodynamics of vesicle adhesion, studying the location and order of the adhesion transition and drawing an analogy to wetting. Subsequently, we discuss the role of spatially extended membrane-substrate interactions and buoyancy on the vesicle shape by studying the effective contact angle and the modification of the transversality condition. Both geometric features are often used in experiments to extract thermodynamic parameters. The transition from an adsorbed, upward-buoyant vesicle to an unbound vesicle is discussed next. The paper concludes with an adsorption diagram, showing the adsorption transition and the spinodal of the metastability of an adsorbed, upward-buoyant vesicle.

## Model and Methods

**Figure 2:**
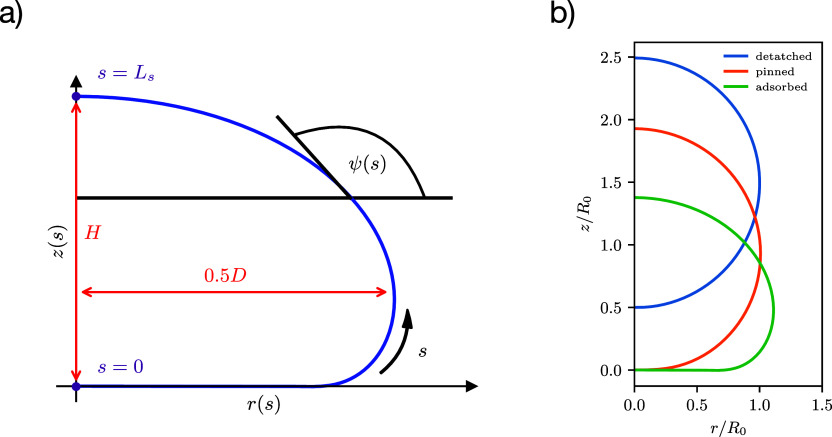
(a) Axially symmetric vesicle shape is described by the arc length, *s*, starting at the bottom center of the vesicle with *s* = 0 and the tangential angle to the arc, ψ(*s*). These coordinates can easily be translated to the cylindrical coordinates, *z*(*s*) and *r*(*s*). The height *H* and diameter *D* determine the vesicle eccentricity. (b) Illustration of an exemplary adsorbed, pinned, and detached vesicle. The pinned vesicle is within the range of the potential, however, is hardly deformed. Note that there is no thermodynamic transition between pinned and adhered state; for any spatially extended membrane-substrate potential, it is a gradual crossover.

To investigate the static properties of adsorbed, buoyant vesicles, we consider axially symmetric shapes. These shapes are described using the contour length *s* and tangential angle ψ(*s*),^5,6,18,28,29^ as depicted in Figure 2a. ψ(*s*) can easily be transformed to cylindrical coordinates using
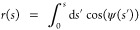
1
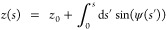
2

The description via the arc length enables us to numerically minimize the vesicle’s energy, by varying the coefficients of the following Fourier expansion^11,18,28,29^
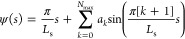
3

The expansion coefficients, *a*_*k*_, vanish for a spherical vesicle. The arc length, *L*_s_, and coefficients need to be chosen to assert *r*(*L*_s_) = 0. The membrane configuration is thus described by {*a*_*k*_}, *z*_0_, and *L*_s_.

The energy of the vesicle is comprised of three contributions: bending energy,^30^ interaction with the substrate,^4−6,15^ and buoyancy. The bending energy is calculated via the Helfrich Hamiltonian, ,^30^ which integrates over the two principle curvatures,  and , and takes the following form^5,6,18,29^
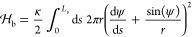
4κ is the bending rigidity and sets the energy scale. As the lipids within the bilayer can flip-flop between the monolayers, both monolayers are identical and the spontaneous curvature of the membrane vanishes. Since the membrane is laterally homogeneous, the Gaussian curvature term only provides a constant contribution and also needs not to be considered.

Whereas prior studies often modeled the interaction between vesicle membrane and substrate per unit area by a contact potential^4−6^ (see ref (10),^12^,^15^,^18^,^31^, for exceptions), we consider long-range potentials of the Hamaker form.
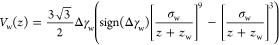
5

The sign function assures that the potential remains repulsive at short distances even for Δγ_w_ < 0.  shifts the minimum of *V*_w_(*z*) to *z* = 0. Note that the long-range power-law decay is scale-free. When exploring the impact of the range of the adhesion potential, we compare the behavior in the long-range potential to that in a short-range potential of the form
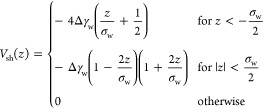
6

Both potentials are illustrated in Figure 1. Integrating the membrane-substrate interaction over the vesicle, we obtain the adhesion energy
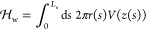
7

We consider a mass-density difference, Δρ, between the liquid enclosed by the vesicle and the surrounding solution, which results in a vertical force. We thus introduce the following term for the gravitational energy
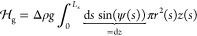
8where *g* denotes the gravitational acceleration constant. Upward buoyancy corresponds to Δρ*g* < 0.

The total vesicle energy is given by the sum of these three contributions,  (Table 1).

**Table 1:** Typical Parameters Used for the Numerical Minimization of the Vesicle Energy

variable	value
*N*_max_	144
*N*_*s*_	288, 576
σ_w_/*R*_0_	0.03, 0.06
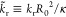	100, 1000
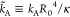	100
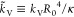	0, permeable membrane

In order to close the vesicle at the top, we restrain deviations of *r*(*L*_s_) from zero by a stiff umbrella potential
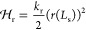
9that is added to the restrained energy.

Unless noted otherwise, we consider that the membrane area, *A*, is conserved.
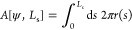
10

Deviations of the membrane area from that of the corresponding area, *A*_0_, of a spherical vesicle with radius, *R*_0_, are penalized by the umbrella potential
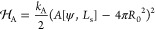
11

The umbrella-potential constants, *k*_r_ and *k*_A_, are chosen large enough such that deviations from the reference values are negligible. These restraints via an umbrella potential can be interpreted as compressibilities.

The volume, *V*, of the vesicle
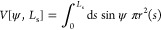
12can also be restrained by adding the term
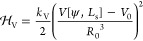
13to the restrained energy. Unless explicitly noted otherwise, we investigate permeable vesicles that can adjust their enclosed volume and set *k*_V_ = 0.

Obviously, one cannot simultaneously fix the membrane area and the vesicle volume to that of a spherical vesicle, *A*_0_ = 4π*R*_0_^2^ and , and observe a deformation from the spherical shape upon adhesion. Either one employs a volume ratio, 3*V*_0_/(4π*R*_0_^3^) < 1, or one conceives the parameters, *k*_A_ and *k*_V_, as the inverse area compressibility of the membrane and the inverse (volume) compressibility of the enclosed fluid. We adopt the latter scheme with  only in the Vesicle Shape and Effective Contact Angle without Buoyancy section.

Measuring all length scales in units of *R*_0_, and all energies in units of κ, we obtain for the membrane tension, γ, and the pressure difference, Δ*P*, across the membrane the dimensionless expressions
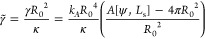
14
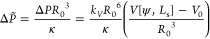
15

To quantify the contact-zone area, we used the following thermodynamic definition^18^
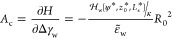
16

In summary, the restrained energy reads
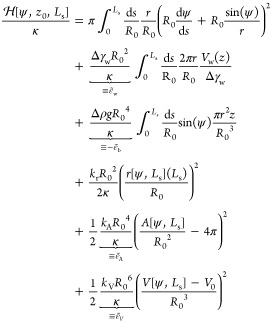
17

The thermodynamic state of the vesicle in contact with an undeformable substrate is characterized by two dimensionless parameter combinations, (4,5) and (7) that measure the relative strength of adhesion and buoyancy with respect to the bending energy, respectively. By the same token,  and  quantify the dimensionless inverse compressibilities of membrane area, *A*, and enclosed volume, *V*, relative to the bending energy.

We minimize this restrained energy numerically by adjusting the Fourier coefficients *a*_*i*_ as well as *L*_s_ and *z*_0_, using a conjugate-gradient-descent method.^18^ is the value of the energy at its minimum, i.e., the free energy in the thermodynamic equilibrium state, characterized by  and . Note that our model does not consider thermal fluctuations, i.e., any additional contributions to the free energy due to Helfrich repulsion,^32^ a softening of the bending rigidity due to thermal fluctuations,^33^ or additional finite-temperature effects^34^ are ignored.^a^

For simplicity, we define the dimensionless energy difference between the vesicles in contact with a substrate and a free, unbound vesicle in the absence of buoyancy, *H* = *H*_b_ = 8πκ. This reduced adsorption free energy takes the form
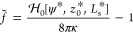
18

## Results and Discussion

### Absence of a Critical, Vesicle-Size-Dependent Adsorption Transition for Spatially Extended Membrane-Substrate Interaction

**Figure 3:**
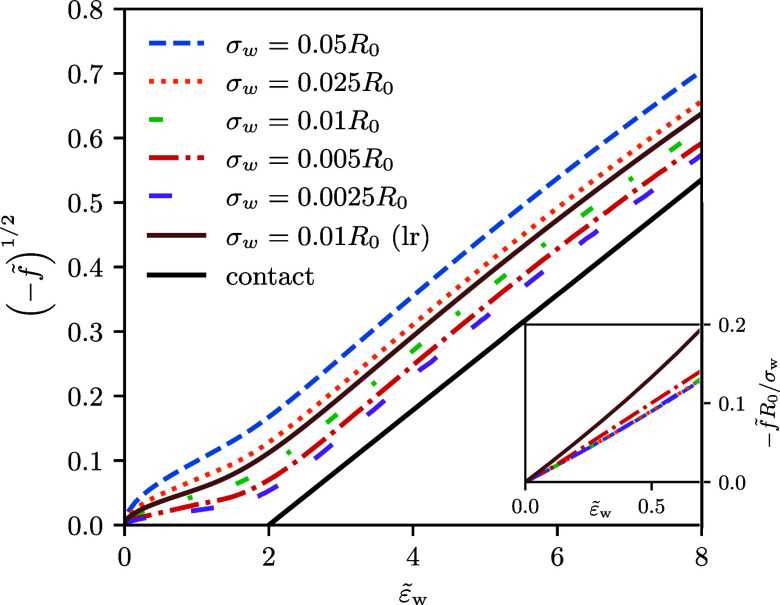
Root of the adsorption free energy, , as a function of the interaction strength, , for the short-range and long-range potentials, *V*_c_ (dashed lines) and *V*_w_ (full line), in the absence of buoyancy, . For the short-range potential, the width, σ_w_, of the membrane-substrate interaction is varied. For a contact potential with σ_w_ → 0, one expects  in the vicinity of the second-order transition, as indicated by the black, solid line. Inset: linear dependence of the adsorption free energy, *f̃*, on  and σ_w_/*R*_0_, as expected in the pinned state, see eq 22.

We first investigate the influence of the spatial range of the potential, on the energy as well as the vesicle shape in the absence of buoyancy, 

In Figure 3, we present the dependence of the root of the reduced adsorption free energy, *f̃*, as a function of the adhesion strength, , without buoyancy, . Immediately, we observe the absence of an adsorption transition, *f̃* = 0, at (4,5,15) as expected for a contact potential. For fixed  and type of potential, the attraction increases with σ_*w*_, i.e., -f̃ increases with σ_w_. The membrane-substrate interaction is qualitatively similar for short-range and long-range potentials. The adsorption free energy, −*f̃*, of the long-range potential with width σ_w_ = 0.01*R*_0_ is slightly higher than that of the short-range potentials with the same width and remains smaller than that of a short-range potential with the increased width, σ_w_ = 0.025*R*_0_. Importantly, the adsorption free energy, −*f̃*, remains positive for all , if the membrane-substrate interaction has a nonzero spatial extent, σ_w_ > 0.

For a contact potential, σ_w_ → 0, we expect a second-order adsorption transition with^4−6^
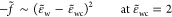
19

Indeed, in the interval , the behavior resembles a linear dependence of  on .^4^ This is indicated by the black, solid line in Figure 3, whose slope has been adopted to the data for small σ_w_ = 0.0025*R*_0_. The free energy of an unbound vesicle, *f̃* = 0, however, is not approached at . Instead, the vesicle adopts a “pinned state”^7,15^ for , where the vesicle remains almost spherical and touches the substrate at a point. In this state, the vesicle can benefit from the spatially extended attraction between membrane and substrate even without deformation (see Figure 2b for an illustration of the different vesicle states).

In this pinned-vesicle regime, , we use Derjaguin’s approximation to obtain an upper bound of the vesicle’s free energy as the free energy of a spherical vesicle interacting with a planar substrate^35^
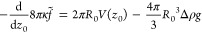
20where *z*_0_ = *z*_cm_ – *R*_0_ is the (closest) distance between the vesicle membrane and the substrate. The minimal free energy is obtained at height, *z*_min_, determined by the condition, . In the absence of buoyancy, we obtain *z*_min_ = −σ_w_/2 for the spatially extended membrane-substrate interaction, *V*_sh_. Integration yields the upper bound
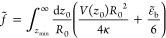
21

In the absence of buoyancy, , we obtain for the short-range potential, *V*_sh_, the bound
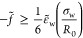
22

The data collapse in the inset of Figure 3 confirms this linear behavior of *f̃* that corresponds to a first-order adsorption transition at . Thus, for any nonzero spatial extent of the membrane-substrate interaction, σ_w_ > 0, the adsorption transition is of first order and occurs at . A critical adsorption transition at  only emerges in the singular limit, σ_w_/*R*_0_ → 0.

The adsorption transition can also be observed by monitoring the contact area of the adsorbed vesicle. There is no shape singularity at the edge of the adhesion zone but the vesicle shape gradually detaches from the substrate.^18^ By the same token, we also do not observe a singularity in the thermodynamic response function, , which would be the signal of a second-order transition.

### Desorption-Free-Energy Profile, *g̃*, as a Function of the Vesicle’s Center of Mass—Analogy to Wetting

In order to rationalize the thermodynamics of vesicle adsorption, we find it useful to draw an analogy to the wetting behavior of laterally uniform, liquid films on a substrate.^19−23^ In wetting, one considers a laterally homogeneous liquid film of area *L*^2^ with density profile ρ(*z*) (with *z* being the coordinate normal to the substrate) that coexists with its vapor and is in contact with a solid substrate. The detailed density profile, ρ(*z*), is obtained by minimizing a free-energy functional, , that plays a similar role as the restrained energy  in eq 17. Rather than characterizing the wetting film by the details of the density profile, ρ(*z*), one often adopts a coarse-grained description, characterizing the system configuration solely by the film thickness, *z*_int_, or position of the liquid–vapor interface. Given the detailed density profile, we define the film thickness via the integral criterion, *z*_int_ = *Z*_int_[ρ] ≡ ∫_0_^∞^d*z*[ρ(*z*) – ρ_v_]/[ρ_l_ – ρ_v_], where ρ_v_ and ρ_l_ denote the coexistence densities of the liquid and vapor, respectively. The type of wetting transition can be classified by the shape of the interface potential, , where  minimizes  under the restraint *z*_int_ = *Z*_int_[ρ]. The interface potential quantifies the free energy per unit area of a liquid–vapor interface at a distance, *z*_int_, away from a substrate. The minimization of *g*(*z*_int_) with respect to *z*_int_ yields the equilibrium film thickness. A liquid film wets the substrate if *g* adopts its minimum at *z*_int_ = ∞.

Phenomenologically, the interface potential, *g*(*z*_int_), is comprised of short-range and long-range contributions. The short-range contribution of the interface potential decays exponentially with *z*_int_ and arises from the distortion of the liquid–vapor interface due to the presence of the substrate. The long-range contribution stems from van-der-Waals interactions and exhibits a power-law dependence, *g*_lr_ = *A*/*z*_int_^2^.^b^ The latter contribution always dominates for large *z*_int_, and wetting is only possible if the long-range contribution does not oppose large film thicknesses, *z*_int_. In particular, if the short-range contribution favors wetting, the wetting transition occurs when the long-range contribution changes sign, *A* = 0. In this case, the wetting transition is of first order.

We adopt the analog description for the desorption of a vesicle: rather than characterizing the vesicle by the details of its shape, ψ(*s*), *z*_0_, *L*_s_, we adopt a coarse-grained description, characterizing the system configuration solely by the center of mass, *z*_cm_, of the vesicle above the substrate. Given the detailed description, we define the center-of-mass coordinate
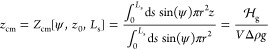
23

Minimizing the energy of the vesicle  under the restraint *z*_cm_ = *Z*_cm_[ψ, *z*_0_, *L*_s_], we obtain the vesicle potential, . In the practical calculation, we mollify the restraint by a narrow Gaussian, adding the umbrella potential
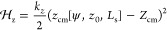
24with strength *k*_z_ to the energy . Let [ψ*,z*,*L*_s_^*^] denote the minimum of  for a given *z*_cm_, we define the desorption-free-energy profile, *g̃*, as a function of *z*_cm_
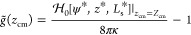
25

The (meta)stable position of the vesicle is obtained by minimizing *g̃*. A vesicle desorbs from the substrate if *g̃* adopts its minimum at *z*_cm_ = ∞. A pinned or adsorbed vesicle, where the minimum of *g̃* occurs at a microscopic *z*_int_ < ∞, corresponds to a partially wet state.  is the analog of the interface potential, *g*(*z*_int_), in wetting phenomena and the correspondence is summarized in Table 2.

**Table 2:**
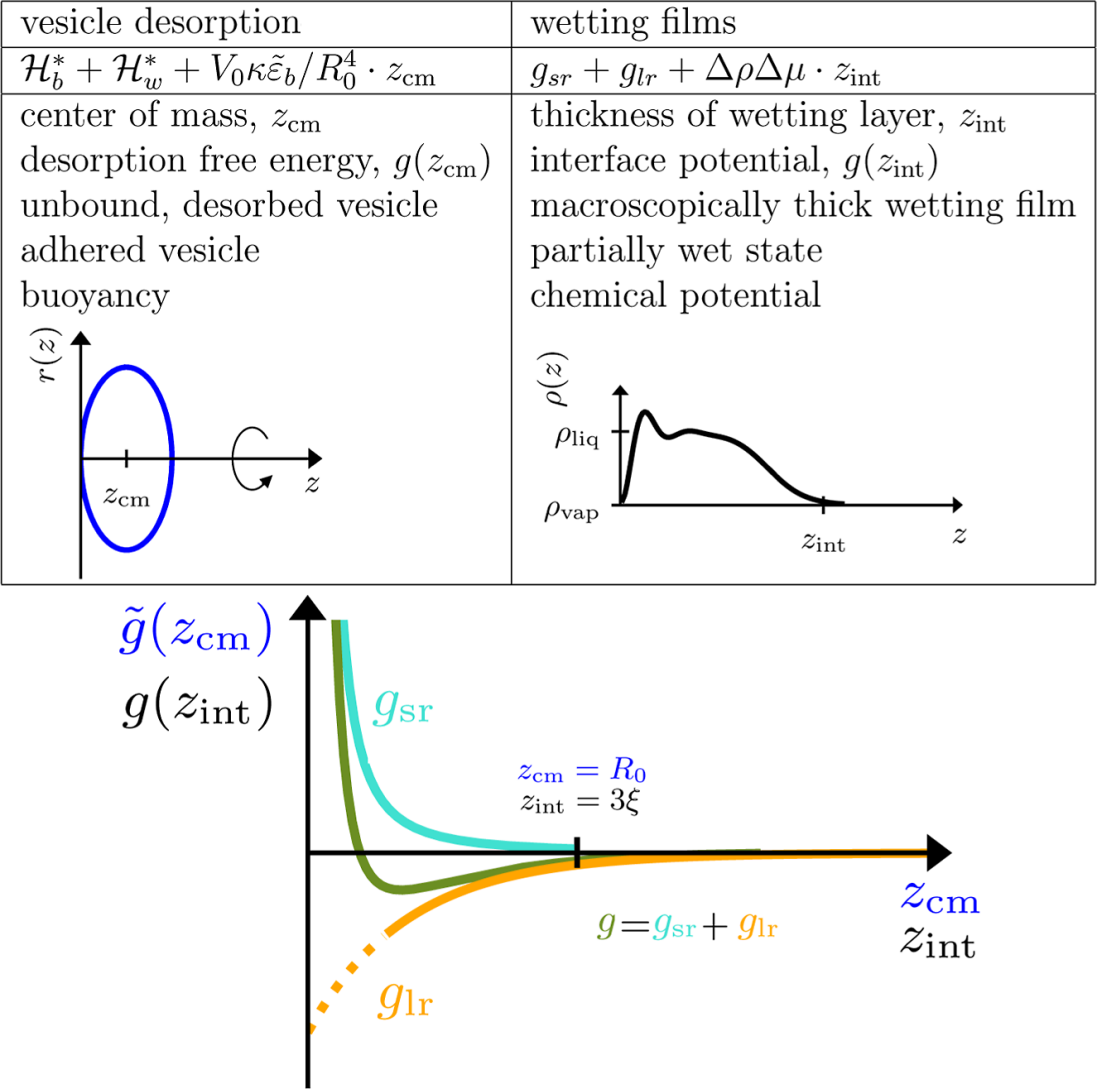
Summary of the Analogy between Vesicle Desorption and Wetting

The desorption-free-energy profile, , comprises a short-range contribution that quantifies the bending energy,  of the vesicle and a longer-range contribution, arising from the membrane-substrate interaction, . Note that the bending energy favors a desorbed, unbound state but rapidly decreases for *z*_cm_ > *R*_0_. In turn, the spatially extended membrane-substrate interaction, , dominates the behavior for *z*_cm_ > *R*_0_.^c^ Thus, the membrane-substrate interaction, , plays a similar role as the long-range contribution of the interface potential in wetting, even if the direct membrane-substrate potential, *V*, does not decay like a power-law.

In particular, since the short-range, bending contribution favors vesicle desorption, the adsorption transition occurs when the longer-range contribution, , changes from repulsive to attractive, i.e., at . This rationalizes why the adsorption transition occurs exactly at  and is a first-order transition.

In the absence of buoyancy, , the desorption-free-energy profile,  vanishes for *z*_cm_ → ∞. Buoyancy of vesicles introduces an additional term in the energy, which is linear in the distance to the substrate.
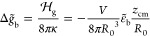
26

Wetting occurs at liquid–vapor coexistence and *g* → 0 for *z*_int_ → ∞. If the chemical potential differs from its coexistence value by Δμ, this difference will give rise to a linear contribution to the interface potential. Therefore, we identify the role of the chemical-potential difference in wetting with the buoyancy, , in vesicle adhesion. Upward buoyancy corresponds to supersaturation in the wetting scenario. In analogy to wetting, the vesicle adsorption transition can only occur at vanishing buoyancy.

There is, however, an important difference between the wetting of a liquid on a substrate and the adsorption of a vesicle. Wetting phenomena refer to singularities of the excess free energy of the substrate that only occur when the lateral extent of the liquid film on the substrate becomes macroscopic (thermodynamic limit). In the case of vesicle adsorption, however, the vesicle is always of finite size, *R*_0_, and the limit *R*_0_ → ∞ is nontrivial as the thermodynamic control variables, adhesion strength , and buoyancy , depend on the vesicle size, *R*_0_. Thus, only in the mean-field approximation, where we minimize *H*[ψ, *z*_0_, *L*_s_] and ignore thermal fluctuations, can a thermodynamic adsorption transition occur.

### Vesicle Shape and Effective Contact Angle without Buoyancy

**Figure 4:**
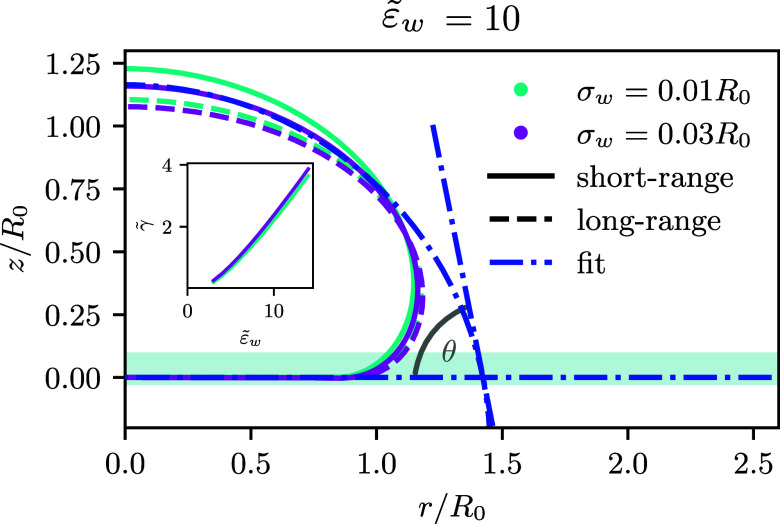
System without volume restraint: vesicle shapes at  for the different potential ranges, as well as short-range and long-range potentials, eqs 6 and 5. The lightly blue shaded area indicates where the potential is more attractive than 1% of its strength, Δγ_w_. The dark blue, dash-dotted lines exemplify how a contact angle, θ (gray), is obtained from a cap fit. The inset presents the dimensionless membrane tension, γ̃, as a function of .

Figure 4 presents the vesicle shape for different interaction ranges, illustrating that the spatial extent of the membrane-substrate interactions exerts an influence on the entire shape of the vesicle, e.g., the maximal curvature at contact and the height of the vesicle.

As we increase the adhesion strength, , the contact area, *A*_c_, increases, and the height of the vesicle, *z*(*L*_s_), decreases. In turn, the dimensionless membrane tension, , increases (see inset of Figure 4) as well. At finite adhesion strength (and thus finite membrane tension), there are two geometric characteristics of the droplet shape that are commonly studied in experiments:

The local shape of the membrane at the edge of the contact zone is determined by a balance of adhesion and bending energy. The transversality condition relates the maximal curvature at the contact zone to the adhesion strength. For a zero-ranged contact potential, one obtains *C*_1max_^2^ = 2Δγ_w_/κ,^4−6^ by minimizing the vesicle energy with respect to the contact-area radius. This local equilibrium balance between adhesion and curvature is a boundary condition for the elastic shape equations and, therefore, it is independent of the vesicle size, *R*_0_.

At high tension, the bending energy can be approximately ignored,^d^ and the vesicle shape on large length scales, i.e., outside the ultimate vicinity of the contact area, is dictated by a balance between adhesion and induced membrane tension—just like in the case of a liquid droplet on a substrate. In the latter case, the lateral variation of the contact point yields Young’s equation^36,37^
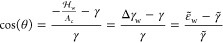
27with an effective contact angle, θ, that can be obtained by fitting the upper part of the vesicle shape by a spherical cap, as indicated in Figure 4. In contrast to liquid droplets; however, (i) the membrane tension γ is not an intrinsic material property of the membrane (like the bending rigidity) but depends on the vesicle shape, and (ii) the change of the effective substrate energy upon contact between membrane and substrate comprises the adhesion energy *and* the membrane tension, −Δγ_w_ + γ.

The crossover between this large-scale, tension-dominated behavior and the local bending-dominated behavior is given by the capillary length, .^14^ Studying large vesicles, this length scale can be identified by the deviation of the vesicle shape from a cap-shaped fit, yielding an estimate for the membrane tension, γ. In conjunction with the measurement of the effective contact angle, θ, these observations provide an estimate for the adhesion energy Δγ_w_ = κ(1 + cos θ)/λ^2^. The validity of such an estimate, however, has to be carefully inspected.

The inset of Figure 4 presents the tension of a permeable membrane, i.e., in the absence of a volume restraint, . The tension remains small, and cos θ > 1 suggests that θ = 0°. This is in qualitative contrast to the value 45° < θ < 90°, obtained by a cap-shaped fit, as shown in the main panel of Figure 4 for . Similar to the geometric determination of the contact angle of liquid drops,^38,39^ the fit focuses on the top of the vesicle, minimizing the influence of the local, bending-dominated behavior in the ultimate vicinity of the edge of the contact zone. Even for the largest adhesion strength studied, , however, the energy associated with membrane tension, 4πκγ̃ = 48.6κ, only becomes comparable to the bending energy, *H*_b_ = 44κ, but does not dominate the behavior. Thus, Young’s equation is not applicable to permeable vesicles, at least for .

Only in this section, we restrain changes in the vesicle volume by using a finite volume compressibility, , and use a large adhesion strength, , in order to increase the adhesion-induced membrane tension. If , both the area and volume of the vesicles are fixed, and the vesicle adopts a spherical shape, independent from the strength of the adhesion. In the following numerical example, we set the dimensionless inverse compressibilities of vesicle area and volume equal, , so that the relative changes of the area and volume from their reference values, *A*_0_ = 4π*R*_0_^2^ and *V*_0_ = 4π*R*_0_^3^/3, are comparable. This introduces an additional dimensionless parameter, , that quantifies the strength of the area and volume restraint in units of the bending energy. In the following, we choose  or 1000, referring to these setups as moderately and strongly restrained vesicles.

**Figure 5:**
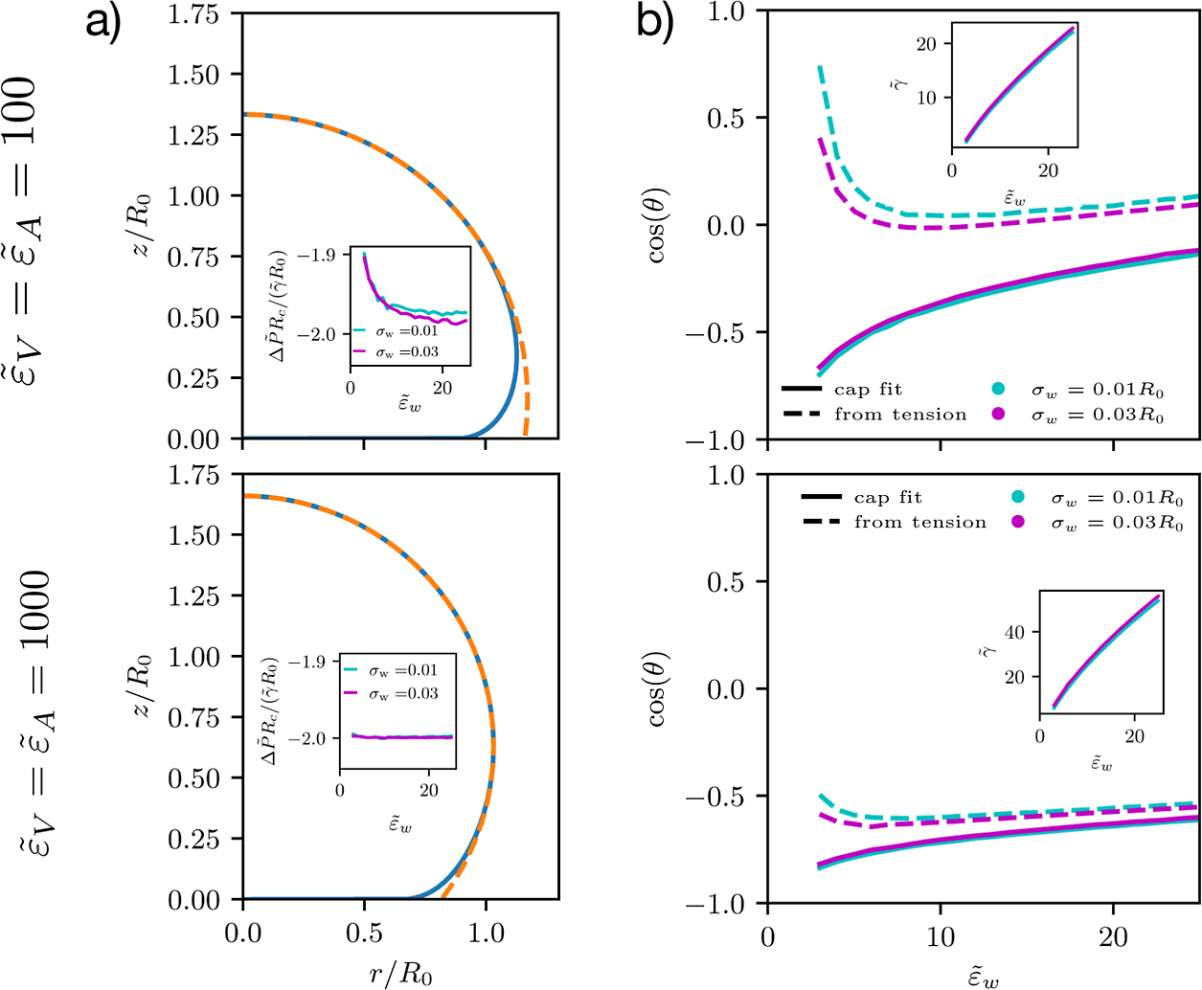
Two systems with volume restraints of  (top row) and 1000 (bottom row): Panel (a) depicts the vesicle shape and corresponding cap fit, representing the associated droplet shape at  and σ_w_ = 0.03*R*_0_. The difference between fit and shape becomes much smaller for large inverse compressibilities. The insets show the ratio . The Laplace equation for a liquid drop yields . Panel (b) compares the contact angles for the same two different strengths of the inverse volume and area compressibilities,  and . Dashed lines present the calculated contact angles, and solid lines are obtained from the spherical-cap fit. The inset shows the tension of the membrane, on which the calculated angle is based.

Figure 5a depicts the vesicle shape and the cap-shaped fit for . Upon increasing the restraint, we can clearly observe that the cap-shaped fit describes a larger portion of the vesicle. This demonstrates that the capillary length, λ decreases, i.e., the induced membrane tension increases with stiffer restraints, . This is confirmed by comparing the insets of panel b), showing the monotonous increase of γ̃ with adhesion strength.

Figure 5b compares the geometrically determined contact angle, θ, with the prediction of Young’s equation, eq 27. With a stiff volume restraint and large adhesion strength, qualitative agreement between the two estimates of the contact angles is achieved. This agreement improves as we increase the adhesion strength and stiffen the restraint.

In the opposite limit of moderate restraint or weak adhesion, however, the prediction of the contact angle via Young’s equation results in a spurious nonmonotonous behavior of cos θ upon an increase of adhesion, i.e., the contact angle exhibits a maximum at  for  and at  for , respectively. Both values, , are well above the adsorption transition even for a contact potential.

Complementary to the capillary length, λ, we can estimate to what extent the energy balance is bending-dominated or tension-dominated by studying the pressure difference, Δ*P̃*, between the vesicle’s inside and outside. In the tension-dominated regime, we expect to recover Laplace’s equation  for liquid drops, where *R*_c_ denotes the curvature radius of the spherical-cap fit. The ratio  is plotted in the inset of panel a. The deviations for the moderately restraint system, , highlight again the remaining role of the bending energy. Upon increasing the adhesion strength or the strength of the restraint, we recover Laplace’s equation, as expected for a tension-dominated vesicle.

To summarize, the concept of Young’s equation is only applicable to vesicles if the adhesion-induced tension dominates the bending energy. This condition is difficult to meet for it requires (i) very strong adhesion strengths, which exceed the adsorption threshold of a contact potential by an order of magnitude, and (ii) a stiff restraint of the enclosed vesicle volume, i.e., a virtually impermeable vesicle. Hence, the transversality condition appears to be more robust for accurately extracting the adhesion strength from the vesicle shape.

### Role of Buoyancy

Buoyancy is of importance as, in experimental setups, it is regularly used to let vesicles sediment onto the substrate. The balance between buoyancy and bending is quantified by the dimensionless parameter , i.e., buoyancy significantly affects the shape of large vesicles. In experiments, for instance, buoyancy arises if the vesicle is filled and surrounded by two different aqueous solutions with a density difference Δρ. Due to the exchange of water across the membrane, however, the vesicle volume can adapt.

#### Vesicle Shape and Transversality Condition

Our simulations show, as intuitively expected, that downward buoyancy, , increases the radius, *r*_c_, of the edge of the contact zone and reduces the height, *z*(*L*_s_), of the top of the vesicle. A heavy vesicle flattens nonuniformly, i.e., downward buoyancy gives rise to a nonuniform rescaling of the height coordinate, *r*(*s*), *z*(*s*), compared to . Upward buoyancy, , in turn, leads to nonuniform stretching normal to the substrate.

**Figure 6:**
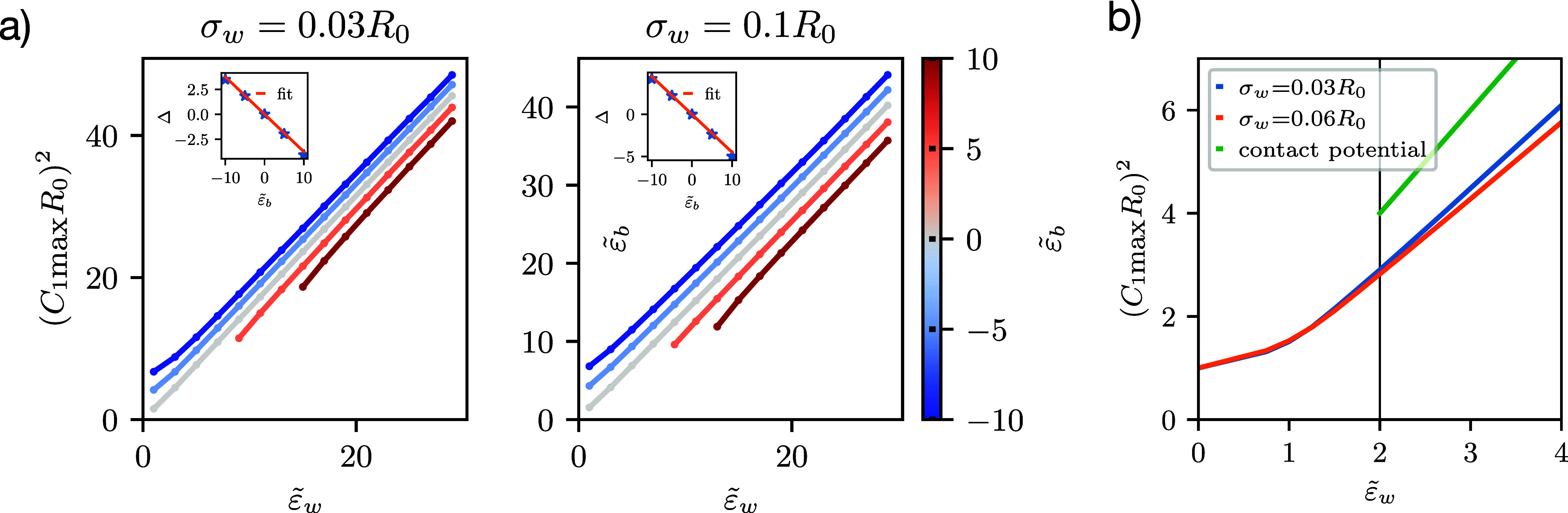
(a) Transversality condition as a function of adhesion strength,  for different buoyancy values, . The left and right panels correspond to two distinct ranges of membrane-substrate interaction, σ_w_ = 0.03*R*_0_ and 0.1*R*_0_, respectively. The insets present the shift Δ (see eq 28) as a function of . (b) Small-adhesion limit at  in a long-range potential: the contact curvature goes toward one (spherical vesicle). The green line presents the transversality condition of a contact potential for .

In the following, we focus on the shape in the ultimate vicinity of the edge of the adhesion zone. In the absence of buoyancy, we have found that the transversality condition  holds for zero-ranged and spatially extended membrane-substrate interactions.^18^ In Figure 6a, we plot the square of the dimensionless maximal curvature, *C*_1max_*R*_0_, as a function of the adhesion strength, , for various values of buoyancy, , as indicated by the color code. Even in the presence of buoyancy, the data are still well described by the linear relation in the strong-adhesion limit, (18)
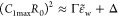
28

While the slope, Γ, is chiefly set by the interaction range σ_w_/*R*_0_, we now observe an offset, Δ, that mainly depends on buoyancy. This buoyancy dependence of the offset is depicted in the insets of Figure 6a for two values of the membrane-substrate interaction ranges, σ_w_/*R*_0_ = 0.03 and 0.1, respectively. For , this offset vanishes, and it decreases with buoyancy. Δ is well parametrized by a linear dependence, i.e.,  for the interval of buoyancy considered. The proportionality constant depends on the functional form of the membrane-substrate interaction, and we find −0.375 and −0.45 for σ_w_/*R*_0_ = 0.03 and 0.1, respectively. Δ effectively accounts for the modification of the interplay between adhesion and bending due to gravity. Since the transversality condition is a local balance at the edge of the contact zone, we hypothesize that the relevant length scale is the range of the membrane-substrate interaction.

The coefficient Γ in eq 28 depends on the range of the potential, with Γ decreasing with increasing σ_w_/*R*_0_.^18^ Throughout the remainder of this section, we will focus on the low-adhesion limit, . Here, the modified transversality condition, eq 28, does not hold (see Figure 6b). In the case of a contact potential, the vesicle would not adhere but remain spherical and in the bulk for . For a membrane-substrate interaction of nonzero spatial extent, the vesicle could gain energy from the attractive potential without deformation—pinned state (see Figure 2b).^15^ It will deform just slightly to optimally balance adhesion and bending energy, which leads to  for . At vanishing adhesion strength, , and buoyancy , the spherical vesicle is nonadsorbed and remains in the bulk.

If a vesicle is subjected to upward buoyancy, it reduces its energy by increasing the distance to the substrate, i.e., the thermodynamically stable state is desorption. The adsorbed state of an upward-buoyant vesicle, however, will remain metastable if there exists a free-energy barrier, , that needs to be overcome to proceed from the adsorbed to the desorbed state.

#### Desorption-Free-Energy Profile and Its Hysteresis

**Figure 7:**
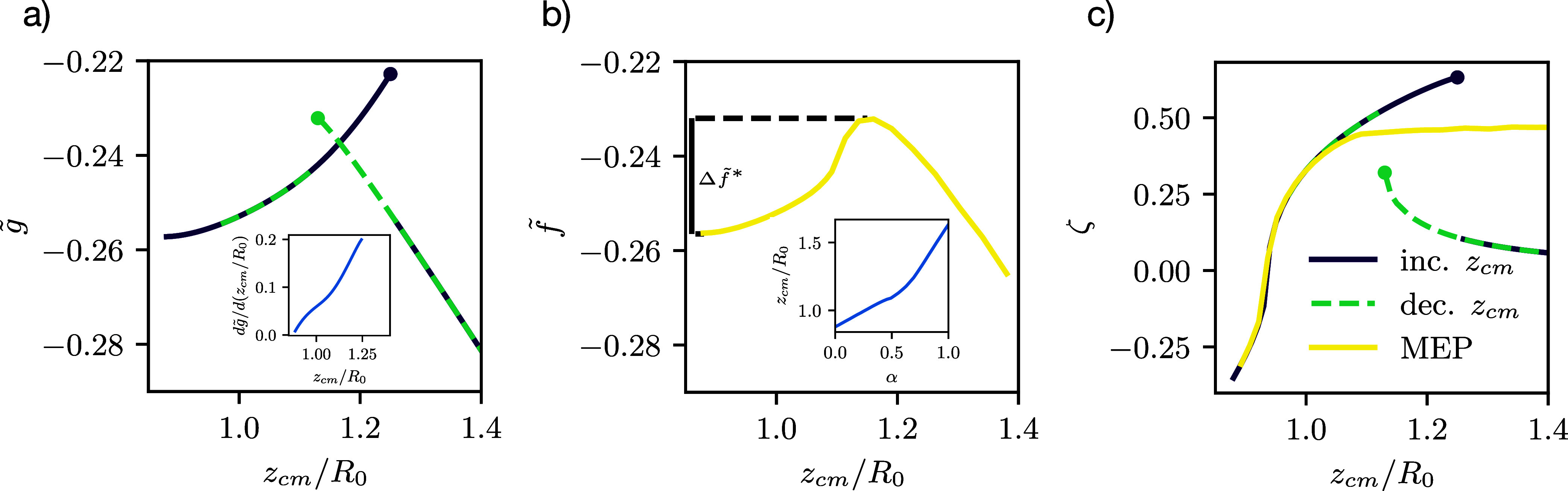
(a) Study of the adhesion behavior using an additional term, restraining *z*_cm_, with *g* the corresponding free energy (see eq 25). Depending on whether the vesicle’s center of mass is slowly increased (blue) or decreased (green) a hysteresis can be observed. (b) The transition path from adsorbed to desorbed using an MEP. As this method finds the optimal path, no hysteresis will occur. The free-energy barrier  is indicated on the left. (c) The eccentricity, ζ of the vesicles for the transitions in panel a is shown, highlighting the difference in vesicle shapes between these pathways. All systems are at  and .

Restraining the vesicle’s center of mass, *z*_cm_, we can either increase *z*_cm_, starting from the adsorbed state, or decrease the center-of-mass height, *z*_cm_, starting with a desorbed, spherical vesicle. Figure 7a shows the desorption-free-energy profile for increasing (blue) and decreasing (green) *z*_cm_. We clearly observe a hysteresis when using *z*_cm_ as the control parameter: Upon increasing *z*_cm_ from the adsorbed state, the vesicle initially remains adhered to the substrate but vertically elongates into a prolate shape. In this adhered, deformed state, the vesicle benefits from the substrate attraction but its bending energy increases with *z*_cm_. Then, at the blue point, this deformed adsorbed state becomes unstable and, at fixed *z*_cm_, the vesicle discontinuously changes to a more spherical shape with a larger minimal distance, *z*_0_, from the attractive substrate.

We characterize the vesicle shape by its signed eccentricity
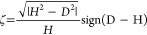
29where *H* = *z*(*L*_s_) – *z*_0_ and *D* = 2 max_s_[*r*(*s*)] denote the height and diameter of the vesicle, respectively (as indicated in Figure 2a). For oblate shapes, *H* < *D*, the eccentricity is negative, whereas prolate shapes yield ζ > 0. Figure 7c quantifies that the adsorbed vesicle is oblate. Upon increasing *z*_cm_, we initially observe that its shape changes from oblate to prolate. Subsequently, the prolate shape of the adsorbed, deformed vesicle becomes unstable, and the vesicle jumps into a more spherical shape at the spinodal limit of the adsorbed, deformed state. Moving farther away from the substrate, the vesicle–substrate interaction, , becomes negligible and the vesicle becomes spherical, ζ = 0, because this shape minimizes .

Conversely, upon decreasing *z*_cm_ along the green curve, Figure 7c shows that the vesicle remains nearly spherical until, at *z*_cm_ ≈ 1.13*R*_0_, it snaps into contact with the attractive substrate.

Thus, restraining *z*_cm_, we cannot reversibly transform an adsorbed vesicle into a desorbed one or vice versa, i.e., *z*_cm_ is not a suitable reaction coordinate (aka control parameter) to estimate the free-energy barrier between the adsorbed and the desorbed state.

#### MEP—Reversible Desorption Mechanism

In order to find a reaction coordinate (aka control parameter), α, that describes the reversible transformation from an adsorbed to a desorbed vesicle, to estimate the concomitant energy barrier, , and provide insights into the desorption mechanism, we use the MEP.^24−27,40−42^

According to Sec. Model and Methods, we describe the vesicle by the dimensionless parameters, , that we compile into a vector. A (meta)stable state or saddle point of the vesicle is characterized by the vanishing of the dimensionless chemical-potential vector .

A discretized path of vesicle configurations is defined by a sequence of vesicle states, **P**_*i*_ with *i* = 1, ..., *S*_max_ = 31. The contour parameter along the path is calculated by adding the distance between neighboring vesicle states
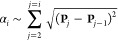
30and α is normalized such that α_*S*_max__ = 1. We interpolate the discretized path **P**_*i*_ at α_*i*_ to a continuous path **P**(α)—the string—using a cubic spline for each component of **P**.^24^

The MEP is then defined by the condition that the chemical potential, μ_⊥_ = **μ** – (**μ**·**t**)**t**, perpendicular to the path with tangent vector **t** = (d**P**/dα)/|d**P**/dα| vanishes for all 0 ≤ α ≤ 1.^24^

We obtain this path by the improved string method^24^^e^ that iterates a two-stage cycle: First, each vesicle configuration is updated according to **P**_*i*_ → **P**_*i*_ – **μ**_⊥_δ with δ = 10^–5^.^f^ Subsequently, the states are uniformly distributed along the path, i.e., **P**_*i*_ = **P**[α_*i*_ = *i*/(*S*_max_ – 1)]. The iteration of cycles is ended if ∑_*i*_|**μ**_*i*⊥_| is sufficiently small, .^g^

The starting state, α = 0, of the MEP is the (meta)stable adsorbed vesicle. For upward buoyancy, however, there exists no (meta)stable ending state; the vesicle can continuously reduce its energy by increasing *z*_cm_. Thus, the ending point of the path will be a vesicle whose *z*_cm_ increases with each iteration cycle. To avoid this extension of the path, we remove states with *z*_cm_ > 1.5*R*_0_ and use the spline reparameterization to uniformly redistribute the states.

In Figure 7b, we present the normalized free energy, *f̃*, along the MEP. In order to compare the results to the desorption–free-energy profile, , we parametrically plot *f̃*(α) versus *z*_cm_(α). The latter quantity is shown in the inset. In the vicinity of the minimum, the adsorbed state, g̃, and *f̃* agree, indicating that *z*_cm_ is an appropriate control parameter. In the *z*_cm_-interval, where restraining *z*_cm_ yields two solutions, however, the MEP provides a reversible mechanism of desorption with a well-defined free-energy barrier, . The energy and the shape of the vesicle vary continuously with α, as illustrated by the eccentricity in panel c. Note that the vesicle will eventually adopt a spherical shape along the MEP for *z*_cm_ → ∞.^h^

**Figure 8:**
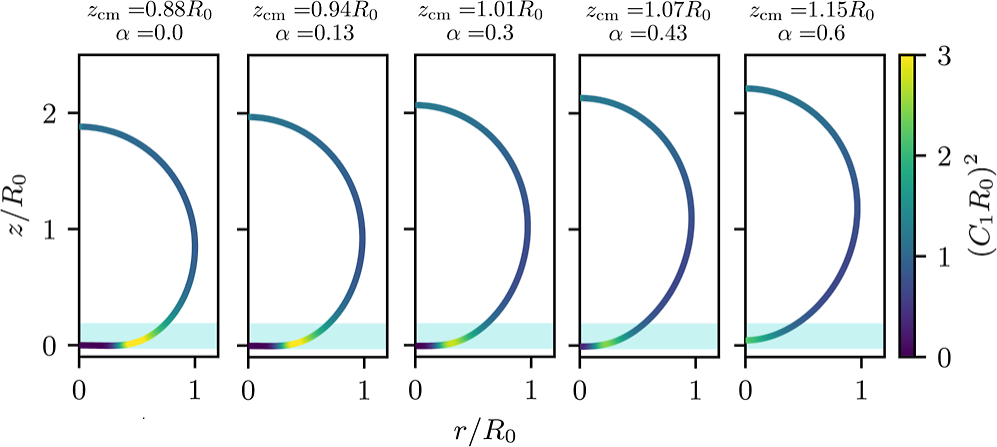
Vesicle shapes along the MEP of the reversible desorption process at  and . The color code indicates the curvature  along the vesicle. The turquoise area indicates the zone, at which the potential decreases by 1/30.

The shape of the vesicle along the MEP is shown in Figure 8. From left to right, α and *z*_cm_ increase, and the contact area, *A*_c_, decreases. We also observe that the maximal curvature that occurs at the edge of the contact zone, decreases as the vesicle becomes prolate.

#### Metastable Adsorption of Upward-Buoyant Vesicles

At strong adhesion and weak upward buoyancy, an adsorbed vesicle will be metastable, i.e., the adsorbed state is a local minimum of  that is separated by an energy barrier, , from the global minimum—the desorbed state, *z*_cm_ → ∞. Thus, desorption is a thermally activated process, and metastable adsorbed vesicles can be observed in experiments for a finite time.

Upon decreasing the adhesion or increasing the buoyancy, the energy barrier, , between the metastable adsorbed and the stable desorbed state will decrease and vanish at the spinodal, . At this line in the plane—the spinodal of the adsorption diagram—desorption occurs spontaneously, and metastable adsorbed vesicles cannot be experimentally observed for 

The hysteresis of the desorption–energy profile, , allows us to approximately extrapolate toward the limit of metastability. The spinodal of the adsorption diagram corresponds to the observed loss of metastability of the adsorbed state at  without restraint on *z*_cm_. The instability of the adhered, deformed vesicle upon increasing *z*_cm_, marked by the blue point in Figure 7a, signals the loss of metastability of the adsorbed state at  but subjected to an applied upward force  (see inset), caused by the positional restraint. Using eq 26, we can approximately substitute the upward restraint force via an increase of the buoyancy force, i.e., . Assuming that the vesicle volume remains *V* = 4π*R*_0_^3^/3, we have to increase the upward buoyancy by . For the system parameters studied in Figure 7, this approximate extrapolation yields the estimate  for one spinodal point of the adsorption diagram.

**Figure 9:**
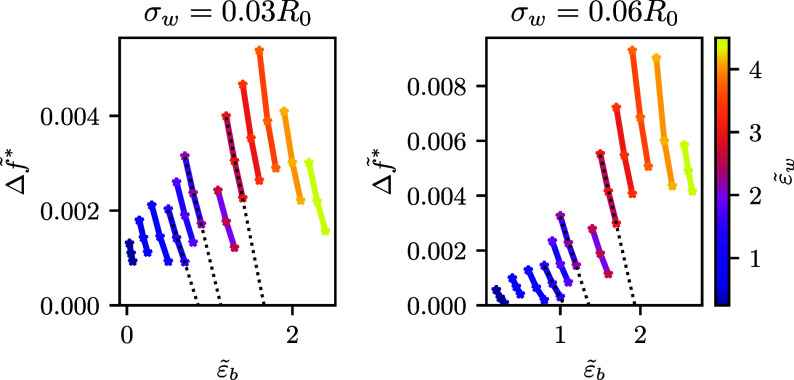
Height of free-energy barrier,  obtained by the MEP. Thick lines correspond to constant adhesion strength, , as indicated by the color code in the key. For exemplary data sets, dotted black lines visualize the extrapolation used to estimate the spinodal, where the free-energy barrier vanishes, .

In the following, we investigate the spinodal of the adsorption diagram by accurately calculating the free-energy barrier, , of the MEP for adhesion strengths , i.e., covering the pinned state and moderate adhesion strengths. At fixed ,  decreases as we increase the upward buoyancy, . Linearly extrapolating  versus  to zero, we accurately determine the spinodal line, , of the adsorption diagram. This procedure is illustrated in Figure 9 for two different ranges, σ_*w*_, of membrane-substrate interaction. For every , we considered three MEPs with an energy barrier smaller than 20% of the normalized energy, f̃, of the metastable state at , , in order to perform the extrapolation, .

**Figure 10:**
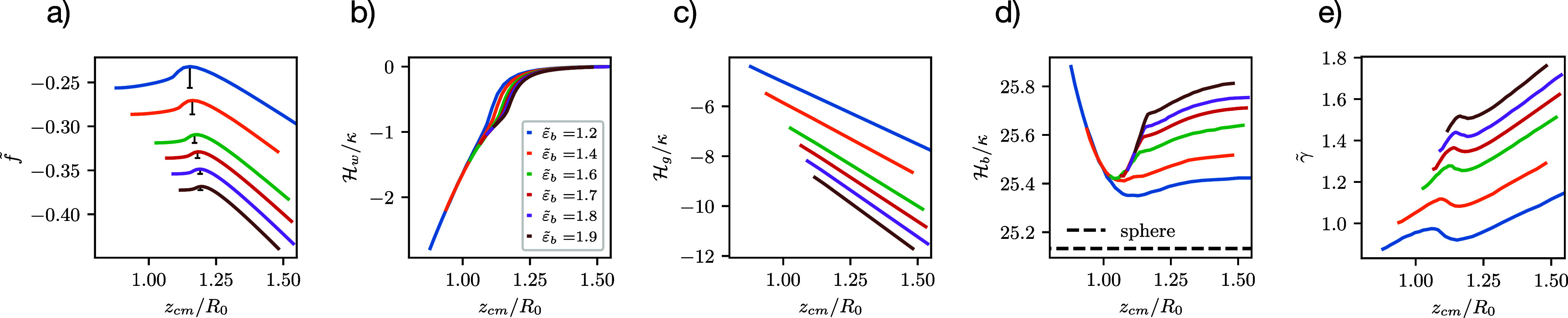
(a) Variation of normalized free energy, *f̃*, along MEPs at  and various values of upward buoyancy, , as indicated by colors. The black bars indicate the height of the free-energy barrier, . Panels (b–d) present the three energy contributions, adhesion, bending, and gravitational energy, respectively. Panel (e) shows the membrane tension along the MEPs.

The dependence of the MEP on buoyancy and the concomitant interplay between bending, adhesion, and gravitational energy is illustrated for  in Figure 10. First, we discuss the dependence of the metastable, adsorbed state (α = 0, i.e., left starting point of the MEP) on upward buoyancy, . Upon increasing  at fixed adhesion strength, the energy of the metastable, adsorbed state chiefly decreases because its center of mass, *z*_cm_, lifts up, reducing the energy, , in the gravitational field (also cf. panel c). This effect outweighs the loss of attractive interaction of the metastable, adsorbed vesicle with the substrate, i.e., the reduction of the thermodynamically defined contact area, *A*_c_^th^, upon an increase of upward buoyancy, . The shape change of the adsorbed vesicle slightly decreases the bending energy, , i.e., the vesicle shape becomes more spherical as  increases (see panel d). This results in a slight increase of the vesicle volume, as suggested by the larger negative slope of . These shape changes, however, only contribute little to the total energy.

Second, Figure 10a shows that the shape of the parametric free-energy profile, , along the MEP, remains qualitatively unaltered. For the smallest buoyancy, the vesicle shapes and their eccentricity have been presented in Figures 8 and 7c, respectively. As expected, the free-energy barrier, , decreases with buoyancy, and also the change of *z*_cm_ between the metastable, adsorbed state, and the barrier state decreases. The change in adhesion energy is initially linear (see panel b), and  then approaches zero for the vesicle leaving the range of the membrane-substrate potential for large *z*_cm_. Upon increasing *z*_cm_ along the MEP, we also observe that  linearly decreases (see panel c), indicating that the vesicle volume remains approximately constant along an MEP at fixed  and . In accord with Figure 8, the contact area shrinks as *z*_cm_ increases at small, fixed , and the vesicle shape changes from oblate to prolate along the MEP, giving rise to a minimum in .^i^ For  close to the spinodal, the metastable vesicle, α = 0, already adopts a more spherical shape with smaller bending energy and larger vesicle volume than those at smaller . For these large values of , the vesicle shape becomes more prolate and the bending energy slightly increases with *z*_cm_ along the studied portion of the MEP.

The vesicle transformations can also be observed by the membrane tension shown in panel e. At α = 0, the tension increases with buoyancy. Along the respective MEPs, the tension increases at first, then decreases and reaches a minimum at the free-energy barrier. For higher *z*_cm_, the tension increases approximately linearly. The membrane tension is low for all MEPs as the volume is not restrained.

**Figure 11:**
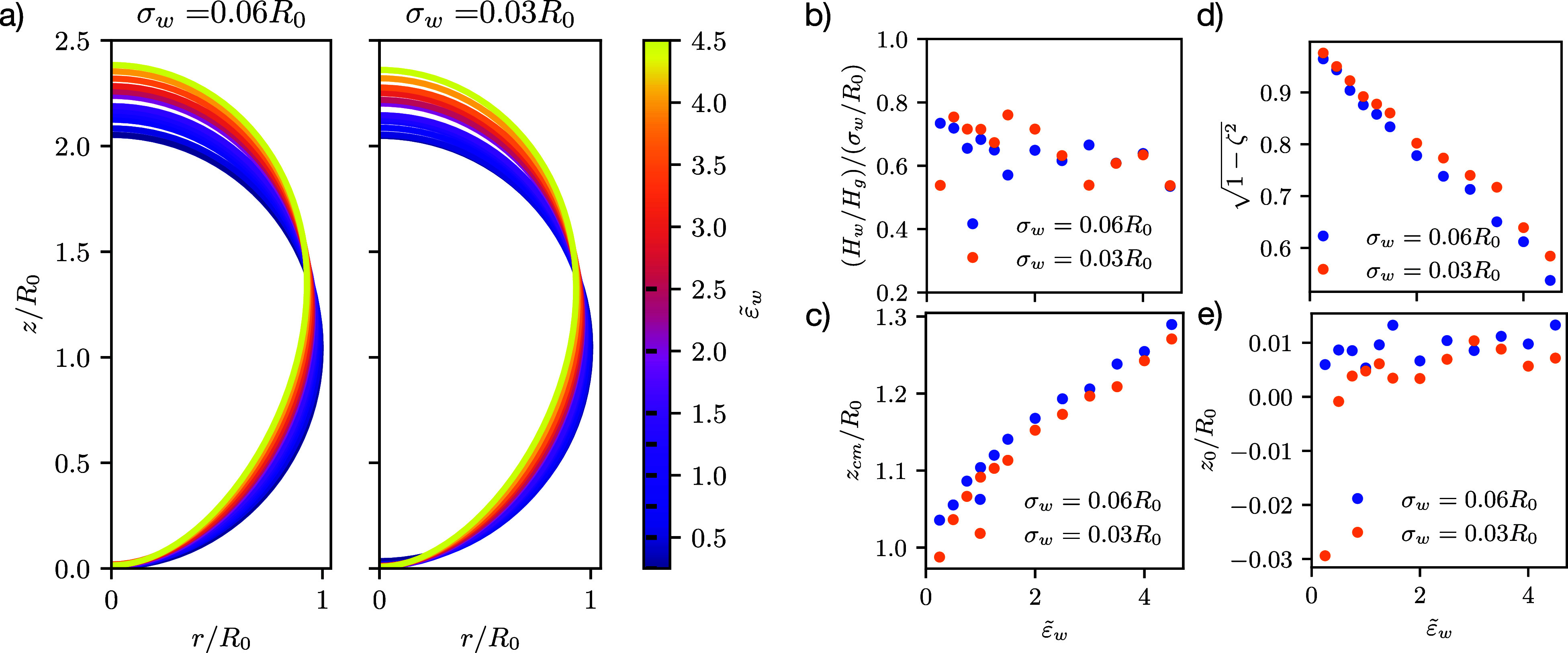
(a) Vesicle shapes at the spinodal for σ_w_ = 0.06 and σ_w_ = 0.03. (b) Ratio of adhesion and gravitational energy (c) increase of the vesicle center of mass, *z*_cm_, (d) decrease of the vesicle’s eccentricity, ζ, and (e) *z*_0_ upon increasing  along the spinodal.

In Figure 11, we explore the vesicle shape along the spinodal as a function of adhesion strength, . Panel a presents the shapes at the barrier of the MEP for the lowest  investigated, whereas the data in the other panels have been linearly extrapolated toward the spinodal. As we increase the adhesion strength, , the spinodal occurs at larger upward buoyancy (cf. Figure 9), the vesicle shape becomes more prolate, and its center-of-mass height, *z*_cm_, increases.^j^

Whereas these characteristics monotonously vary with  along the spinodal, interestingly, the ratio  of adhesion and gravitational energy remains approximately constant along the spinodal. This observation suggests that the location of the spinodal is mainly dictated by the interplay between adhesion and gravitational energy, and it is in accord with Figure 10, where we have also observed that the variation of the bending energy is much less pronounced than the changes of adhesion and gravitational energy. Moreover, the minimal distance, *z*_0_, remains almost constant along the spinodal.

#### Adsorption Diagram

**Figure 12:**
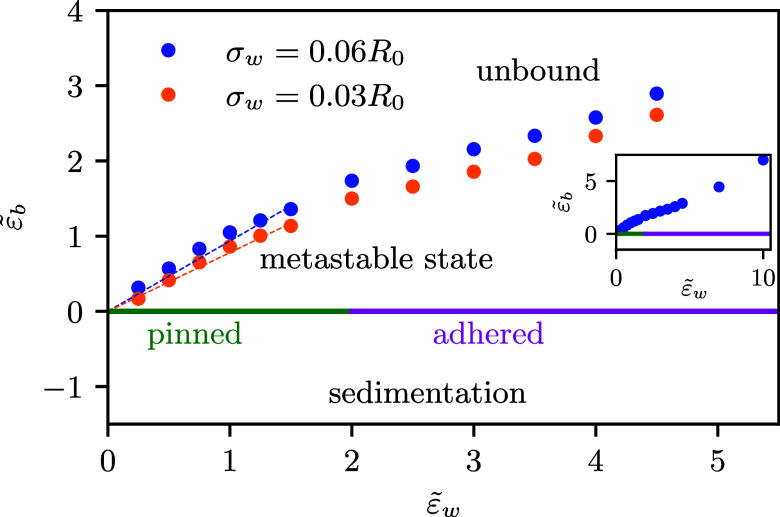
In the presence of buoyancy, we do not only have pinned or adhered vesicles. In downward buoyancy, vesicles sediment. Given a long-range potential and upward buoyancy, the stable state is an unbound vesicle. However, the vesicle can also be in a metastable state, adhering to the substrate. The blue and orange lines present the spinodal stability limit of this metastable state for two different potential widths σ_w_ = 0.06*R*_0_ and σ_w_ = 0.03*R*_0_, respectively.

We summarize our findings in the adsorption diagram, presented in Figure 12. For downward buoyancy, , the vesicle sediments onto the substrate. The membrane-substrate interaction modulates the distance, *z*_0_, between the vesicle and substrate but even for a repulsive substrate, the vesicle is bound to the substrate, i.e., *z*_0_ < ∞.

For zero buoyancy, we observe a first-order adhesion transition at . For a repulsive substrate, , the vesicle desorbs and explores the entire volume of the solution above the substrate. For a weakly attractive substrate, , the vesicle is in a pinned state. By virtue of the spatial extent of the membrane-substrate interaction, the vesicle can benefit from the attraction without substantial deformation and remains bound to the substrate. For stronger attractions, , the balance between bending and adhesion dictates the shape of the vesicle in this adhered state.^4−6^

For upward buoyancy, , the equilibrium state is an unbound, desorbed vesicle, *z*_0_ → ∞. The adsorbed state of a vesicle may, however, be a local minimum of the energy. The metastability of this adsorbed state terminates at the spinodal. This limit of metastability, , is also presented in Figure 12 as a function of adhesion strength, , for two ranges of membrane-substrate interaction. The spinodal buoyancy, up to which the pinned or adhered vesicle remains metastable at the substrate and can be studied by experiment, increases with the adhesion strength, . The data indicate an approximately linear increase of the necessary spinodal adhesion  with buoyancy, as indicated by the lines for small . The inset of Figure 12 includes additional spinodal points at  and , which suggests that the approximately linear dependency extends to stronger adhesion. Also, we observe that a more extended membrane-substrate interaction (larger σ_w_/*R*_0_ at fixed ), stabilizes the metastable vesicle in the vicinity of the substrate.

## Conclusions

We have studied the importance of two, experimentally relevant effects—(i) nonzero spatial extent of interaction between membrane and substrate and (ii) buoyancy—on the adsorption behavior of vesicles. We demonstrated how the spatial extent of the potential changes the second-order adsorption transition at  to a first-order phase transition at . A second-order adsorption transition at (4−6) only emerges as singular limit of the vanishing range of the membrane-substrate interaction, σ_w_/*R*_0_ → 0. We have established an analogy between the adsorption transition of a vesicle and the wetting transition of a liquid film via the desorption-free-energy profile, , as a function of the vesicle’s center of mass and the interface potential, respectively. Vesicle desorption corresponds to wetting, the bending energy is the analog of the short-range contribution of the interface potential and favors desorption or wetting, respectively. The membrane-substrate interaction is the analog of the long-range contribution to the interface potential, even if it exhibits only a finite range. This analogy rationalized why the transition occurs when the membrane-substrate interaction switches from repulsive to attractive.

Buoyancy is the analog of the difference of the chemical potential from the liquid–vapor coexistence value. A proper adsorption transition only occurs at zero buoyancy. For downward buoyancy, vesicles sediment onto the substrate; for upward buoyancy, vesicles desorb. In the latter case, however, adhered vesicles may remain metastable.

Since the dimensionless variable that accounts for buoyancy, , rapidly increases with vesicle size, *R*_0_, buoyancy effects are particularly relevant for large vesicles. Importantly, the size dependence of the dimensionless variables,  and , allows for changing the characteristics of the vesicle merely by changing the vesicle size without altering the chemical details of the membrane-substrate interaction or the solution properties.

Whereas nonzero interaction range and buoyancy alter the qualitative thermodynamics of the adsorption transition, they only modulate the transversality condition, eq 28, that linearly relates the maximal curvature at the edge of the contact zone to the adhesion strength.^4,5^ The slope is determined by the range of the potential, while buoyancy gives rise to an offset in the transversality condition.

For large adhesion strength, adhered vesicles are strongly deformed, and one expects the shape of the vesicle to be dominated by the interplay between membrane tension, γ, and adhesion, Δγ_w_, except for the ultimate vicinity of the edge of the adhesion zone, whose extent is characterized by the capillary length, . The balance between adhesion and induced membrane tension gives rise to an effective Young equation, which has been employed to analyze experiments.^14^ Without volume conservation, however, the membrane tension remains rather small, and we could not identify a region where an effective Young equation accurately approximates the vesicle shape. Strongly restraining volume and area changes, the membrane tension increases upon increase of . Only for extraordinarily strong restraints and large adhesion strength, however, does the Young equation provide an appropriate quantitative description.

The two effects—(i) nonzero spatial extent of interaction between membrane and substrate and (ii) buoyancy—commonly occur in experimental settings and alter the qualitative adsorption thermodynamics and the quantitative description of the vesicle shape. Thus, they have to be accounted for to accurately measure membrane and substrate properties from the shape of an adhered vesicle, for example, by using the adapted transversality condition.

## Footnotes

a Although we do not consider shape fluctuations of the vesicle, the bending and adhesion energies of the self-assembled vesicle are free energies in a thermodynamic sense.

b The interface potential takes the generic form  with *a* and *A* being constants. The characteristic length scale, ξ, of the short-range contribution, is on the order of the correlation length in the liquid or the width of the liquid–vapor interface. *A* is proportional to the Hamaker constant. The functional form of the long-range contribution, *g*_*lr*_, is obtained by integrating the van-der-Waals interaction between substrate and liquid under the assumption of a square-well density profile. Thus, it is only valid for *z*_int_ ≫ ξ.

c In the idealized case of a spherical vesicle in the pinned state (relevant in the vicinity of the adsorption transition), the deformation energy vanishes for *z*_cm_ > *R*_0_, whereas the contribution of the spatially extended membrane-substrate interaction, , extends to *z*_cm_ < *R*_0_ + σ_w_/2 for *V*_sr_.

d For κ = 0, Young’s equation is applicable.^4^

e This method is similar to the nudged-elastic-band approach,^43^ which has been used, e.g., in wetting to determine wetting energies from microscopic models.^40−42^ However, while the string method uses a cubic spline to redistribute the vesicle configurations, the nudged-elastic-band approach connects neighboring vesicle configurations by springs (introducing the spring constant as an additional parameter) and projects the spring force onto the tangent vector, **t**, of the MEP, which is obtained by a finite-difference scheme along the path.

f More generally, we can update the dimensionless configuration vector via  with a symmetric, positive mobility matrix, , and alter the definition of the MEP to  being parallel to **t**. The mobility matrix does not affect the saddle point configurations, and we simply choose .

g The (downward part of the) MEP corresponds to a sequence of states that can be obtained by minimizing the energy  via model-A dynamics, , up to a reparameterization, α(*t*), of the path contour because the model-A dynamics asserts that the path tangent, , is parallel to the chemical potential **μ** at all times, *t*.

h For upward buoyancy, , there exists no stable state with **μ** = 0. Instead, the height, *z*_0_, of the vesicle above the substrate steadily increases because the *z*_0_-component of the chemical potential  remains negative. For *z*_0_ → ∞, the energy is dominated by the gravitational contribution, , where the first term dominates. Thus, for our model without volume restraint, the asymptotic shape is spherical ( for all but the *z*_0_-component) because a spherical vesicle maximizes its volume (and thereby its gravitational energy) at fixed membrane area.

i The minimum of  and the zero-crossing of ζ do not coincide because  is affected by the maximal curvature, *C*_1 max_, at the edge of the contact zone, whereas ζ is less sensitive to *C*_1 max_.

j The shape difference of the vesicle at the instability point along the desorption-free-energy profile and the spinodal arises from the fact that the force,  required to restrain the center of mass, *z*_cm_, and the gravitational force, , act (slightly) differently. This also partly rationalized the discrepancy between the approximate value of the spinodal,  obtained from the desorption–free-energy profile at , and the accurate value, , that we obtained from the MEP, . At the instability point for , the vesicle is more prolate, ζ = 0.63 and *z*_cm_ = 1.25*R*_0_ (see Figure 7c) than at the spinodal along the MEP for the same value of  (ζ = 0.47 and *z*_cm_ = 1.2*R*_0_ vide infra Figure 11b). Due to this shape difference, the desorption-free-energy profile, , at  overestimates the restraint energy to render the vesicle unstable and thereby also overestimates the shift .
